# Immediate Postoperative Management of Cardiac Surgery Patients

**DOI:** 10.14797/mdcvj.1274

**Published:** 2023-08-01

**Authors:** Konya Keeling-Johnson, David Baker, Todd Want, Divina M. Tuazon

**Affiliations:** 1 Houston Methodist, Houston, Texas, US

**Keywords:** cardiopulmonary bypass, hemodynamic derangement, postoperative care

## Abstract

Cardiac surgery is quite common in the United States. Outcomes after cardiac surgery are not only dependent on how the surgery went and how the anesthesia care was provided intraoperatively but also on the optimal management in the postoperative critical care setting. It is of paramount importance that the cardiac intensivist has a comprehensive understanding of cardiopulmonary physiology and the sequelae of cardiopulmonary bypass. Most preventable deaths after cardiac surgery have been linked to postoperative problems in the intensive care unit (ICU).^1,2^ Failure to recognize and rescue a patient from potentially reversible complications is a cause of perioperative morbidity and mortality.

Patients who undergo cardiac surgery often present with multiple rapidly changing clinical problems; they are initially unstable with extremely fluid and dynamic clinical status. Postoperative care of these patients requires knowledge of general fundamental concepts of patient care as well as concepts unique to this set of patients. The initial management of these patients as they return from the operating room is critical, because clinical errors at this time can have far-reaching implications. The initial management should begin even before the patient arrives in the cardiovascular intensive care unit (CVICU). It is vital that the cardiac intensivist reviews the chart and notes the type of surgery, indications, preoperative hemodynamic data, comorbid conditions, medications, and allergies.

Upon the patient’s arrival in the CVICU, a careful systematic assessment of the patient begins with obtaining a comprehensive handoff from the surgical and anesthesia team. The cardiac intensivist should ascertain what procedure was done in the operating room and inquire as to any intraoperative events that might impact the patient’s postoperative course. Then, they should physically examine the patient as part of this initial evaluation. During the initial assessment, the intensivist should avoid focusing on any one issue and attempt to get a global picture of the patient’s clinical status. A thorough knowledge of the specific monitoring and drug delivery lines is imperative, as is knowledge of where the drains are placed. Once the initial assessment is complete, specific issues can be identified, prioritized, and addressed.^3,4^

A 67-year-old male with a past medical history significant for hypertension, hyperlipidemia, type 2 diabetes mellitus, ischemic cardiomyopathy with ejection fraction of 30%, multivessel coronary artery disease, and severe aortic stenosis underwent coronary artery bypass grafting and aortic valve replacement. The postoperative course was complicated by difficulty weaning off pump, requiring inotropic support with dobutamine and pressor support with norepinephrine. Per the anesthesia team handoff, postoperative transesophageal echo showed no perivalvular leak and presence of anterior wall hypokinesis. Vital signs in the ICU showed paced rhythm via epicardial leads at 65 beats per minute (bpm), arterial blood pressure (ABP) 106/51, respiratory rate 18, oxygen saturation 100% on the ventilator settings of synchronized intermittent minute ventilation, rate 18, set tidal volume 500, pressure support 10, positive end expiratory pressure 8, and fractional inhaled oxygen 60%. Chest x-ray and electrocardiogram were unremarkable.

Laboratory results were acceptable except for arterial blood gas showing metabolic acidosis and an elevated lactic acid level. Hemodynamic indices revealed cardiac index (CI) 2.1, central venous pressure (CVP) 13, pulmonary artery pressure (PAP) 27/20, and SvO2 (mixed venous saturation) 51%. Within less than an hour of arrival, changes including ABP 90/40, CI 1.8, CVP 7, and SvO2 48% led to increasing the norepinephrine drip rate. After volume infusion, ABP 110/51, CI 2.0, and CVP 12 increased but SvO2 remained < 60%. The epicardial pacing rate was increased to 80 bpm and the CI 2.3 with SvO2 55%. After another volume infusion, the norepinephrine drip was discontinued. The urine output (UO) was only 0.3 cc/kg/hr. The dobutamine drip was increased to show SvO2 68%, CI 3.0, ABP 115/55, and UO 1 cc/kg/hr. There was no significant chest tube drainage at the second hour and the patient was awakened, but his vitals were ABP 95/50, CI 2.5, CVP 12, and SvO2 63%. Norepinephrine was restarted. More volume infusion was given until the ABP improved. After passing a breathing trial, the ventilator was discontinued. At this point his vitals were ABP 125/60, SvO2 65%, CI 2.8, SpO2 99% on oxygen via 3 liters NC. Over the next 12 hours, the dobutamine was weaned off with acceptable SvO2 and CI. The next day, the patient ambulated and chest tubes were removed. He was then transferred out of the ICU.

## Points to Remember

**Figure 1:**
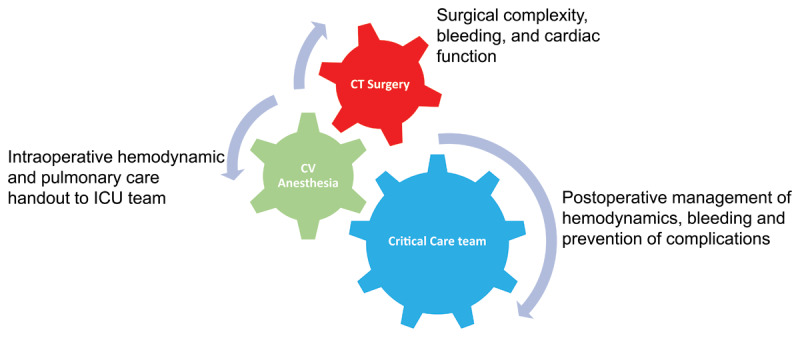
A team effort leads to better outcomes after cardiac surgery. CT: cardiothoracic; CV: cardiovascular; ICU: intensive care unit. Image courtesy of Asma Zainab, MD

- A dedicated staff in the cardiovascular intensive care unit results in better outcomes for the patient. Routine use of a team of intensivist, respiratory therapist, and nurses with experience caring for post cardiac surgical patients results in decreased postoperative mechanical ventilation time, reduced blood product transfusion, shortened hospital length of stay, and decreased total cost.
- A clear understanding of the patient’s preoperative history and intraoperative course is vital to postoperative care. The importance of a comprehensive anesthesia handoff cannot be underestimated.
- It is important to consider what cardiac surgery the patient is undergoing to anticipate changes in hemodynamics that may occur. Valve surgery conveys different hemodynamic problems and concerns postoperatively than coronary bypass surgery. There is a 2- to 4-fold increase in mortality depending on which valve is involved.
- It is important to understand the physiologic consequences of cardiopulmonary bypass (CPB) and to know that there is a transient postoperative ventricular dysfunction that manifests approximately 2 hours after cessation of CPB and is at its worst 4 to 5 hours (or more) after CPB.
- The choice of which, if any, pharmacologic agent to administer requires a complete understanding of the hemodynamic derangement that is present. All cardiac surgical patients are hemodynamically labile after undergoing cardiac surgery. It is important to not overreact to self-limiting hemodynamic swings and appropriately intervene on concerning trends or sudden deterioration. The main goal of hemodynamic management is to maintain adequate organ perfusion and oxygen delivery.
- Close collaboration between the cardiac intensivist, the cardiac anesthesiologist, and the cardiac surgeon is essential for comprehensive postoperative care. This partnership is paramount for the best outcomes for postoperative cardiac patients (Figure 1).
